# Smoking cessation and the risk of cardiovascular disease outcomes predicted from established risk scores: Results of the Cardiovascular Risk Assessment among Smokers in Primary Care in Europe (CV-ASPIRE) Study

**DOI:** 10.1186/1471-2458-13-362

**Published:** 2013-04-18

**Authors:** Pablo Mallaina, Christos Lionis, Hugo Rol, Renzo Imperiali, Andrew Burgess, Mark Nixon, Franco Mondello Malvestiti

**Affiliations:** 1 Primary Care BU Europe, Pfizer, Walton Oaks, Pfizer, Walton Oaks, Dorking Road, Surrey KT20 7NS, UK; 2 Clinic of Social and Family Medicine, Faculty of Medicine, University of Crete, Crete, Greece; 3 Bennebroek Primary Care Center, Bennebroek, The Netherlands; 4 Primary Care Varese ASL, distretto di Tradate, Italy; 5 Real-World and Late Phase Research, Quintiles, Reading, UK

**Keywords:** Cardiovascular, Diseases, Europe, Model, Smoking, Cessation, Framingham, SCORE, Progetto, ASPIRE

## Abstract

**Background:**

Smoking is a major risk factor for cardiovascular disease (CVD). This multicenter, cross-sectional survey was designed to estimate the cardiovascular (CV) risk attributable to smoking using risk assessment tools, to better understand patient behaviors and characteristics related to smoking, and characterize physician practice patterns.

**Methods:**

1,439 smokers were recruited from Europe during 2011. Smokers were ≥40 years old, smoked > 10 cigarettes/day and had recent measurements on blood pressure and lipids. CV risk was calculated using the SCORE system, Framingham risk equations, and Progetto CUORE model. The CV risk attributable to smoking was evaluated using a simulated control (hypothetical non-smoker) with identical characteristics as the enrolled smoker. Risks assessed included CV mortality, coronary heart disease (CHD), CVD and hard CHD. Demographics, comorbidities, primary reasons for consultation, behavior towards previous attempts to quit, and interest in smoking cessation was assessed. Dependence on nicotine was evaluated using the Fagerström Test for Nicotine Dependence. GP practice patterns were assessed through a questionnaire.

**Results:**

The prediction models consistently demonstrated a high CV risk attributable to smoking. For instance, the SCORE model demonstrated that this study population of smokers have a 100% increased probability of death due to cardiovascular disease in the next 10-years compared to non-smokers. A considerable amount of patients would like to hear from their GP about the different alternatives available to support their quitting attempt.

**Conclusions:**

The findings of this study reinforce the importance of smoking as a significant predictor of long-term cardiovascular events. One of the best gains in health could be obtained by tackling the most important modifiable risk factors; these results suggest smoking is among the most important.

## Background

Tobacco is the only legally available consumer product which kills up to one in every two users when used as intended [1]. Smoking kills over 650,000 people in the European Union every year [2]. Smoking is a major risk factor for CVD, and can result in a seven-fold increase in the risk of peripheral arterial disease and at least a two-fold increase in the risk of coronary artery disease [3-5]. The INTERHEART study showed that if a person stopped smoking, they reduced their risk of myocardial infarction by about 65% [6].

Several multi-factorial risk models have been developed to assess the risk for development of CVD, and all of these models include tobacco use as an important predictor, along with age, blood pressure and cholesterol levels, highlighting the impact of smoking on cardiovascular risk. The Framingham Risk Score quantifies the absolute level of CVD risk based on a number of risk factors [7]. In addition to CVD risk, there have been multiple models proposed using the Framingham data to calculate various other absolute risk levels, including CVD mortality, coronary heart disease, intermittent claudication and recurring coronary heart disease [8-12].

In Europe, the Systematic COronary Risk Evaluation (SCORE) system was initiated to develop a risk scoring system for the management of cardiovascular risk in European clinical practice. The SCORE data contains approximately 3 million person-years of observation from 205,178 subjects [13-16].

General practitioners (GPs) in primary care practice play an important role in smoking cessation through measures such as counseling and pharmacotherapy [16]. Research has shown that simple advice provided by GPs can help substantially increase smoking cessation rates [17]. Data from studies on self-reports by GPs show that while the majority of GPs (62% to 98%) question all new patients as to their smoking status, the proportion of GPs offering more intensive interventions or prescribing treatments for cessation is generally lower, and also has shown to differ across and within countries [18-21]. Studies have found that the rates of GPs providing smoking cessation advice vary across European countries [18,19,22,23]. Moreover, GP’s engagement in providing smoking cessation advice to patients depends on factors such as the GP’s smoking status, their attitude towards smoking, their relationship with the patient, and their knowledge of existing smoking cessation interventions [21,24]. Researchers advocate the development of country-specific strategies for encouraging and supporting a GP’s smoking cessation activity [21].

We performed this European, multi-centre, observational, cross-sectional survey of smokers in the primary care setting to provide estimates of the risk of CVD among the smoking population attending primary care in Europe, using standard risk assessment tools. This study also reports on patient behaviors related to smoking as well as physicians practice patterns.

## Methods

We performed this cross-sectional survey involving smokers in the primary care setting, selected from 3 European countries (Greece, Italy and Netherlands). These countries were chosen because they met the study data requirements and provided a geographic spread within Europe in an attempt to create a dispersed distribution. GPs were required to routinely collect lipid levels and blood pressure from smokers as standard practice based on their availabilities, their acceptance to the protocol and informed consent form and their ability to enroll the target population based on their response to their following questions:

•How many smoker patients 40 years or older does the physician see per day/per week/per months?

•Can the investigator enroll 15 smoker patients (who smoke at least 10 cigarettes per day)?

•How long the investigator expect he will need to enroll 15 consecutive smoker patients (who smoke at least 10 cigarettes per day)?

Out of 720 possible sites, 229 met these criteria and were selected.

The final protocol and case report form, any amendments, and informed consent documentation were reviewed and approved by regulatory authorities required in each country.

Participating GPs assessed all smoker patients 40 years or older for their suitability for enrolment into the survey at the time that they attended the centre regardless of their reason for the consultation. Patients were selected consecutively from the patients that visited their doctor on a routine basis irrespective of the reason for their visit. The recruitment period for the survey was flexible to enable a maximum of 15 patients per GP. There was no set time limits for recruitment into this study. Patient recruitment continued until there were sufficient patients (approx 1400) to provide adequate precision for the study endpoints. Informed consent was obtained for those patients who met the study inclusion/exclusion criteria and who agreed to participate. Suitable patients smoked at least 10 cigarettes per day during the previous month and had blood pressure and lipids level data in their clinical records (within the last 12 months).

Once informed consent was obtained, the investigator administered the survey to patients and completed the study case report form. Demographic information was collected on age, gender, height and weight. Laboratory parameters included total cholesterol, High-density lipoprotein (HDL), Low-density lipoprotein (LDL) and systolic and diastolic blood pressure. Information on medical history was also captured (history of cardiovascular disease, diabetes, hypertension, hypercholesterolemia and chronic obstructive pulmonary disease (COPD)) including reason for consultation and family medical history.

Patients were asked whether they had persistent chronic cough, excess mucous and phlegm, shortness of breath, or wheezing, and whether they were currently being treated for hypertension, hypercholesterolemia, diabetes, or COPD. Parameters on patient behaviors related to smoking included the Fagerström Test for Nicotine Dependence (FTND) [25], years of smoking, previous attempts to quit smoking, previous use of any smoking cessation programs, longest time without smoking, interest in quitting smoking, and interest in type of smoking cessation support. Practice patterns related to smoking behavior included asking the patient whether they were provided with smoking cessation advice and whether any behavioral or pharmacotherapy support was offered.

Information about the clinic was also collected, including number of practitioners, patient volume and physician smoking status. To assess typical clinical practice patterns related to smoker behavior, we asked the clinician whether typically in their practice smoking cessation advice, behavior support or pharmacotherapy is offered to patients who have an interest in quitting smoking.

### Statistical analysis

Continuous variables were summarized using mean and standard deviation (SD), and categorical variables were summarized using counts and proportions. 95% confidence intervals were calculated where appropriate.

For smokers, 10-year cardiovascular risk was calculated using multiple tools and models including various Framingham risk scores, SCORE, and the CUORE risk score [8-13]. The cardiovascular disease risk scores used in this study are summarized in Table 1. To compare the predicted probability of a cardiovascular event calculated using these scores against those for non-smokers; we re-calculated each risk score after re-classifying each smoker as a non-smoker. Risk differences were calculated by taking the difference of the predicted probabilities between smokers and non-smokers. Relative risk estimates were calculated by dividing the predicted probability for smokers by the non-smokers.

**Table 1:** **Description of study endpoints and associated risk score**, **CV ASPIRE study**, **Europe**, **2011**

**Risk score**	**Study endpoint**	**Study population**	**Study sample size**	**Risk period**	**Predictors**
SCORE^a^	Cardiovascular Mortality Risk	Smokers attending primary care in Europe; age 39 to 91 years.	1419	10 years	Age, gender, systolic blood pressure, total cholesterol
Framingham^b^	Coronary Heart Disease Risk	Smokers attending primary care in Europe; age 39 to 74 years.	1275	10 years	Age, gender, diabetes status, systolic and diastolic blood pressure, total cholesterol, LDL cholesterol
Framingham^c^	Cardiovascular Disease Risk	Smokers attending primary care in Europe; age 39 to 91 years.	1368	10 years	Age, gender, diabetes status, systolic blood pressure, treatment of hypertension, total cholesterol, LDL and HDL cholesterol
Framingham^d^	Hard Coronary Heart Disease Risk	Smokers attending primary care in Europe; age 39 to 79 years.	1372	10 years	Age, gender, systolic blood pressure, treatment of hypertension, total cholesterol, HDL cholesterol
Framingham^e^	Intermittent Claudication Risk	Smokers attending primary care in Europe; age 45 to 84 years.	1161	4 years	Age, gender, systolic blood pressure, treatment of hypertension, total cholesterol, coronary heart disease, number of cigarettes smoked per day
Framingham^f^	Recurring Coronary Heart Disease Risk	Female smokers attending primary care in Europe; age 39 to 74 years, with history of coronary heart disease.	68	2 years	Age, gender, diabetes status, systolic blood pressure, fasting total cholesterol, fasting HDL cholesterol
Progetto CUORE^g^	Risk of Coronary or Cardiovascular Event	Smokers attending primary care in Europe; age 39 to 69 years.	1117	10 years	Age, gender, diabetes status, systolic blood pressure, total cholesterol, HDL cholesterol, treatment of hypertension

References:

a: Conroy RM, Pyörälä K, Fitzgerald AP, et al., SCORE project group. Estimation of ten-year risk of fatal cardiovascular disease in Europe: the SCORE project. Eur Heart J 2003; 24(11):987–1003.

b: Wilson PW, D'Agostino RB, Levy D, et al. Prediction of coronary heart disease using risk factor categories. Circulation 1998;97(18):1837–1847.

c: D'Agostino RB, Sr., Vasan RS, Pencina MJ, et al. General cardiovascular risk profile for use in primary care: the Framingham Heart Study. Circulation 2008; 117:743 753.

d:. D'Agostino RB, Sr., Grundy S, Sullivan LM, et al. Validation of the Framingham coronary heart disease prediction scores: results of a multiple ethnic groups investigation. JAMA 2001; 286(2):180–187.

e: Murabito JM, D'Agostino RB, Silbershatz H, et al. Intermittent claudication. A risk profile from the Framingham Heart Study. Circulation 1997;96(1):44–49.

f: D'Agostino RB, Russell MW, Huse DM, et al. Primary and subsequent coronary risk appraisal: new results from the Framingham Study. Am Heart J 2000;139(2 Pt 1):272–281.

g: Palmieri L, Panico S, Vanuzzo D, et al. La valutazione del rischio cardiovascolare globale assoluto: il punteggio individuale del Progetto CUORE. Annali dellIstituto superiore di sanita 2004:40(4):393–399.

All analyses were performed using SAS® Version 9.2 (Cary, NC).

## Results

### Participating sites and participants

A total of 1439 subjects were enrolled in the study (Greece n=571; Italy n=443, and Netherlands n=425) from 104 sites. A summary of site demographics is shown in Table 2.

**Table 2:** **Site demographics by Country**, **CV ASPIRE study**, **Europe**, **2011**

**Characteristic**	**Greece (N=38)**	**Italy (N=31)**	**Netherlands (N=35)**	**Total (N=104)**
Mean number of practitioners at site (range)	7.2 (1–20)	1.7 (1–6)	1.7 (1–5)	3.8 (1–20)
Mean typical patient volume per day (range)	127.4 (25–400)	28.0 (7–80)	42.5 (20–180)	71.1 (7–400)
Mean participating physician years of practice (range)	18.2 (10–35)	29.5 (11–44)	22.5 (5–40)	22.9 (5–44)
Participating physician smoking status, n (%)				
Smoker	0 (0.0%)	2 (6.9%)	2 (6.5%)	4 (4.1%)
Ex-smoker	12 (31.6%)	13 (44.8%)	11 (35.5%)	36 (36.7%)
Non-smoker	26 (68.4%)	14 (48.3%)	18 (58.1%)	58 (59.2%)
Mean total number of smoker patients 40 years or older screened to enroll 15 study patients (range)	27.0 (15–70)	23.8 (4–50)	22.5 (3–50)	24.7 (3–70)
Median time taken to enroll 15 patients (days), (range)	20.0 (3–90)	20.0 (0–365)	79.5 (3–365)	25.0 (0–365)
Mean number of patients that met inclusion/exclusion criteria but did not show interest in participating in study (range)	3.6 (0–15)	8.4 (0–30)	26. (0–15)	4.7 (0–30)

Descriptive characteristics of participants. On average, 3.8 practitioners per site enrolled patients and the mean patient volume per day was 71.1.

Patients from Greece were, on average younger, than those from Italy or Netherlands; patients from Greece had a mean age of 55.6 years, Italy 58.2 years and Netherlands 59.2 years (Table 3). The most common reason for consultation was general medical examination (45.3%), followed by a cardiovascular related reason (16.2%). The primary reason for consultation varied across countries: for Greece and Italy General medical examination was the most frequent reason (57.2% and 57.1%, respectively), for Netherlands this was 16.1%, with the most frequent reason being Cardiovascular related (31.7%). Similarly, a higher proportion of patients in the Netherlands (13.1%) visited their GP for pulmonary related conditions compared to Greece (8.8%) or Italy (4.9%). Mean fasting total cholesterol, HDL and LDL values at baseline were 5.4 mmol/L, 1.3 mmol/L and 3.4 mmol/L, respectively. The mean total cholesterol and LDL were both above the recommended optimal level for the general population (European Cholesterol Guidelines (2003), optimal cholesterol <5.0 mmol/L, optimal LDL <3.0 mmol/L). Mean systolic blood pressure was 135.5 mmHg and diastolic blood pressure 81.6 mmHg, which are both above the normal limits (European Cholesterol Guidelines (2007): normal systolic blood pressure: 120–129 mmHg, normal diastolic blood pressure: 80–84 mmHg).

**Table 3:** **Patient characteristics of smokers by Country**, **CV ASPIRE study**, **Europe**, **2011**

**Characteristic**	**Greece (N=571)**	**Italy (N=443)**	**Netherlands (N=425)**	**Total (N=1439)**
Mean age, years (age range)	55.6 (39–91)	58.2 (39–85)	59.2 (40–85)	57.4 (39–91)
Gender, n (%)				
Male	352 (61.5%)	263 (58.8%)	199 (46.7%)	814 (56.3%)
Female	220 (38.5%)	184 (41.2%)	227 (53.3%)	631 (43.7%)
Mean BMI, kg/m^2^ (range)	27.6 (12–54)	26.0 (17–43)	27.5 (16–54)	27.1 (12–54)
Primary reason for seeking medical advice, n (%)				
General medical examination	319 (57.2%)	249 (27.1%)	64 (16.1%)	629 (45.3%)
Cardiovascular related	68 (12.2%)	31 (7.2%)	126 (31.7%)	225 (16.2%)
Psychiatric related	15 (2.7%)	14 (3.2%)	20 (5.0%)	49 (3.5%)
Pulmonary related	49 (8.8%)	21 (4.9%)	52 (13.1%)	122 (8.8%)
Gastrointestinal related	34 (6.1%)	34 (7.9%)	12 (3.0%)	80 (5.8%)
Metabolic related	46 (8.2%)	29 (6.7%)	37 (9.3%)	112 (8.1%)
Prescription	22 (3.9%)	6 (1.4%)	1 (0.3%)	29 (2.1%)
Other	12 (2.2%)	57 (13.2%)	89 (22.4%)	158 (11.4%)
Lipid levels				
Mean total cholesterol, mmol/L (SD)	5.7 (1.19)	5.4 (1.27)	5.2 (1.19)	5.5 (1.24)
Mean HDL cholesterol, mmol/L (SD)	1.3 (0.38)	1.3 (0.37)	1.3 (0.59)	1.3 (0.45)
Mean LDL cholesterol, mmol/L (SD)	3.7 (1.05)	3.3 (0.89)	3.2 (1.10)	3.4 (1.04)
Blood pressure				
Systolic blood pressure, mmHg (SD)	135.5 (18.65)	130.9 (14.59)	140.4 (17.42)	135.5 (17.50)
Diastolic blood pressure, mmHg (SD)	82.5 (9.70)	78.7 (8.24)	83.2 (10.63)	81.6 (9.75)
Medical history, n (%)				
Cardiovascular diseases	97 (17.0%)	69 (15.6%)	137 (32.2%)	303 (21.1%)
Diabetes	100 (17.5%)	74 (16.7%)	130 (30.6%)	304 (21.1%)
Hypertension	353 (61.8%)	192 (43.3%)	238 (56.0%)	783 (54.4%)
Hypercholesterolemia	372 (65.1%)	161 (36.3%)	193 (45.4%)	726 (50.5%)
Chronic obstructive pulmonary disease	81 (14.2%)	88 (19.9%)	114 (26.8%)	283 (19.7%)
Currently treated with medication (yes), n (%)				
Diabetes	88 (15.4%)	65 (14.7%)	107 (25.2%)	260 (18.1%)
Hypertension	280 (49.0%)	184 (41.5%)	205 (48.2%)	669 (46.5%)
Hypercholesterolemia	240 (42.0%)	97 (21.9%)	153 (36.0%)	490 (34.1%)
Chronic obstructive pulmonary disease	48 (8.4%)	49 (11.1%)	86 (20.2%)	183 (12.7%)
Family medical history, n (%)				
Cardiovascular diseases	205 (35.9%)	100 (22.6%)	129 (30.4%)	434 (30.2%)
Diabetes	168 (29.4%)	77 (17.4%)	91 (21.4%)	336 (23.3%)
Hypertension	280 (49.0%)	138 (31.2%)	107 (25.2%)	525 (36.5%)
Hypercholesterolemia	205 (35.9%)	76 (17.2%)	70 (16.5%)	351 (24.4%)
Chronic obstructive pulmonary disease	66 (11.6%)	23 (5.2%)	59 (13.9%)	148 (10.3%)
Number of medical conditions, n (%)				
No conditions	105 (18.4%)	145 (32.7%)	75 (17.6%)	325 (22.6%)
1 condition	136 (23.8%)	126 (28.4%)	107 (25.2)	369 (25.6%)
2 condition	181 (31.7%)	96 (21.7%)	100 (23.5%)	377 (26.2%)
3 condition	100 (17.5%)	43 (9.7%)	81 (19.1%)	224 (15.6%)
4 condition	40 (7.0%)	28 (6.3%)	48 (11.3%)	116 (8.1%)
5 condition	9 (1.6%)	5 (1.1%)	14 (3.3%)	28 (1.9%)
Other disorder characteristics, n (%)				
Persistent chronic cough	214 (37.6%)	138 (31.7%)	121 (28.5%)	473 (33.1%)
Excess mucous and phlegm	115 (20.3%)	64 (14.7%)	101 (23.8%)	280 (19.6%)
Shortness of breath	217 (38.2%)	104 (23.9%)	106 (25.0%)	427 (29.9%)
Wheezing	95 (16.8%)	45 (10.3%)	67 (15.8%)	207 (14.5%)

### Comorbid conditions

The most commonly reported medical history categories were hypertension (54.4%) and hypercholesterolemia (50.5%), the majority of whom were currently receiving medication for their disease (85.4% for hypertension, 67.5% for hypercholesterolemia) (Table 3). 33.1% of patients reported persistent chronic cough. Excess mucous and phlegm, shortness of breath and wheezing were also reported by 19.6%, 29.9% and 14.5% of patients, respectively. 48.7% of patients reported at least 1 COPD related disorder. Results differed across countries. Cardiovascular diseases, diabetes and COPD were more common in Netherlands (32.2%, 30.6%, and 26.8% of patients, respectively) compared to Greece (17.0%, 17.5%, and 14.2% of patients, respectively) and Italy (15.6%, 16.7%, and 19.9% of patients, respectively). A higher number of patients were being treated with medication for diabetes and COPD in the Netherlands (25.2% and 20.2% of patients, respectively) compared to Greece (15.4% and 8.4% of patients, respectively) and Italy (14.7% and 11.1% of patients, respectively).

### Cardiovascular risk assessments

Results of the cardiovascular risk assessments based on the prediction scores are shown in Table 4.

**Table 4:** **Cardiovascular risk assessments of smokers**, **CV ASPIRE study**, **Europe**, **2011**

**Cardiovascular risk**	**Risk score**	**n**	**Smokers (a) (N=1439)**	**Hypothetical non-smokers (b) (N=1439)**	**Risk difference (a-b)**	**Risk ratio (a-b/b)**
10-year Cardiovascular Mortality Risk, mean % (range)	SCORE	1419	4.0% (0%-26%)	2.0% (0%-14%)	2.0%	100%
10-year Coronary Heart Disease Risk, mean % (range)	Framingham	1275	17.9% (1%-84%)	11.9% (1%-66%)	6.0%	50%
10-Year Cardiovascular Disease Risk, mean % (range)	Framingham	1368	21.2% (2%-30%)	16.0% (1%-30%)	5.1%	32%
10-Year Hard Coronary Heart Disease Risk, mean % (range)	Framingham	1372	13.2% (0%-49%)	8.0% (0%-48%)	5.2%	65%
4-Year Intermittent Claudication Risk, mean % (range)	Framingham	1161	2.8% (0%-36%)	1.5% (0%-20%)	1.3%	87%
2-Year Recurring Coronary Heart Disease Risk (amongst females with history of CHD), mean % (range)	Framingham	68	6.4% (2%-21%)	4.5% (1%-15%)	1.8%	40%
10-Year Risk (%) of Coronary or Cardiovascular Event, mean % (range)	Progetto CUORE	1117	10.3% (0%-77%)	6.2% (0%-58%)	4.2%	68%

The SCORE model for 10-year cardiovascular mortality risk, predicts a 100% increase among smokers (4%) attending primary care in the participating European countries compared to the risk had they been non-smokers (2%).

The Framingham model for 10-year coronary heart disease risk, predicts a 50% increase among smokers (17.9%) attending primary care in the participating European countries compared to the risk had they been non-smokers (11.9%). The Framingham model also predicts a 32% increase (from 16.0% to 21.2%) in the 10-year CVD risk. For the 10-year hard coronary heart disease risk, the Framingham model predicts a 65% increase (from 8.0% to 13.2%). According to the model, smokers in Greece showed a slightly higher (15%) risk attributable for hard coronary heart disease to smoking (72%) compared to those in Italy (57%) and 11% higher than Netherlands (61%). The 4-year intermittent claudication risk using the Framingham model is predicted to increase by 87% (from 1.5% to 2.8%).

For the Framingham 2-year recurring coronary heart disease risk model, the analyses population is patients with at least one coronary heart disease event. As smoking status is not included in the model for males, the estimated risk for males were not calculated. For females with a previous coronary event, the model predicts in smokers a 40% increase (from 4.5% to 6.4%) in the 2-year recurring coronary heart disease.

The Progetto model for 10-year risk of a coronary or cardiovascular event, predicts a 68% increase (from 6.2% to 10.3%).

### Patient behaviors related to smoking

The mean (SD) FTND score for all patients was 5.0 (2.40) (Table 5). The mean FTND score was higher in Greece (5.7, 95% Confidence interval (CI): 5.5, 5.9) compared to Italy (4.5, 95% CI: 4.3, 4.8) and Netherlands (4.7, 95% CI: 4.5, 4.9).

**Table 5:** **Patient behaviors related to smoking by Country**, **CV ASPIRE study**, **Europe**, **2011**

**Characteristic**	**Greece (N=571)**	**Italy (N=443)**	**Netherlands (N=425)**	**Total (N=1439)**
Fagerström total score, n (%)	552	421	402	1375
Low dependence on nicotine	104 (18.8%)	146 (34.7%)	116 (28.9%)	366 (26.6%)
Moderately dependent on nicotine	239 (43.3%)	179 (42.5%)	205 (51.0%)	623 (45.3%)
Highly dependent on nicotine	209 (37.9%)	96 (22.8%)	81 (20.1%)	386 (28.1%)
Number of years smoking, mean (SD)	31.3 (11.90)	35.5 (12.00)	40.0 (12.21)	35.2 (12.53)
Previously attempted to quit smoking, n (%)	568	441	419	1428
Yes	392 (69.0%)	274 (62.1%)	338 (80.7%)	1004 (70.3%)
No	176 (31.0%)	167 (37.9%)	81 (19.3%)	424 (29.7%)
Number of previous attempts to quit, mean (SD)	2.7 (2.50)	3.0 (3.18)	3.0 (2.87)	2.9 (2.83)
Previous cessation support methods used, n (%)	544	404	396	1344
None	449 (82.5%)	328 (81.2%)	254 (64.1%)	1031 (76.7%)
Nicotine replacement therapy	56 (10.3%)	47 (11.6%)	98 (24.7%)	201 (15.0%)
Behavioral therapy	15 (2.8%)	17 (4.2%)	9 (2.3%)	41 (3.1%)
Alternative medicine	20 (3.7%)	19 (4.7%)	30 (7.6%)	69 (5.1%)
Prescription medicine	29 (5.3%)	6 (1.5%)	35 (8.8%)	70 (5.2%)
Other	5 (0.9%)	11 (2.7%)	17 (4.3%)	33 (2.5%)
Currently interested in quitting smoking, n (%)	562	438	416	1416
Yes	468 (83.3%)	280 (63.9%)	290 (69.7%)	1038 (73.3%)
No	94 (16.7%)	158 (36.1%)	126 (30.3%)	378 (26.7%)
Interest shown in the following cessation support methods, n (%)	537	326	348	1211
None	177 (33.0%)	118 (36.2%)	101 (29.0%)	396 (32.7%)
Nicotine replacement therapy	59 (11.0%)	47 (14.4%)	81 (23.3%)	187 (15.4%)
Behavioral therapy	122 (22.7%)	85 (26.1%)	43 (12.4%)	250 (20.6%)
Alternative medicine	24 (4.5%)	30 (9.2%)	18 (5.2%)	72 (5.9%)
Prescription medicine	212 (39.5%)	92 (28.2%)	114 (32.8%)	418 (34.5%)
Other	12 (2.2%)	10 (3.1%)	33 (9.5%)	55 (4.5%)

It was observed that 70.3% of patients had previously attempted to quit smoking, however, only 23% of patients had previously used any smoking cessation programs. Nicotine replacement therapy (15.0%) was the most common, followed by prescription medicine (5.2%) and then alternative medicine (5.1%). The mean number of years smoking was 35.2 years with differences among countries. Furthermore, a relatively higher percentage of patients in Greece (83.3%) were interested in quitting smoking compared with patients in Italy (63.9%) and Netherlands (69.7%).

### Clinical practice patterns

Overall, 65 out of 98 practices (66.3%) typically offer smoking cessation advice to all smokers and the other 33.7% offer advice to some selected smokers (Table 6). Behavioral support was typically offered to all smokers who want to quit in 77.6% of practices, and pharmacotherapy support in 71.4%. In Greece and Netherlands, smoking cessation advice was given to all smokers in a higher percentage of practices (76.3% and 71.0%, respectively) compared to Italy (48.3%). Pharmacotherapy support was offered in a higher percentage of Netherlands (87.1%) and Italy (82.8%) practices compared to Greece (50.0%).

**Table 6:** **Clinical practice patterns by Country**, **CV ASPIRE study**, **Europe**, **2011**

**Clinical practice response**	**Greece (N=38)**	**Italy (N=31)**	**Netherlands (N=35)**	**Total (N=104)**
Typically in your practice, is smoking cessation advice offered to smokers? n (%)	38	29	31	98
Yes to all smokers	29 (76.3%)	14 (48.3%)	22 (71.0%)	65 (66.3%)
Yes to some selected smokers	9 (23.7%)	15 (51.7%)	9 (29.0%)	33 (33.7%)
Not offered	0	0	0	0
Typically in your practice, is any behavioral support offered to all smokers who want to quit smoking? n (%)	27 (71.1%)	22 (75.9%)	27 (87.1%)	76 (77.6%)
Typically in your practice, is any pharmacotherapy support offered to all smokers who want to quit smoking? n (%)	19 (50.0%)	24 (82.8%)	27 (87.1%)	70 (71.4%)

### Physician practice patterns

At the study visit, 1125 out of 1433 patients (78.5%) were provided with smoking cessation advice and 50.8% were offered behavioral support to help them quit smoking (Table 7). Only 23.9% of patients were offered pharmacotherapy. The percentage of patients that were offered behavioral support was similar by country. However, a relatively higher percentage of patients in Greece (87.9%) were provided with smoking cessation advice compared with Italy (73.2%) and Netherlands (71.5%). Whereas, a relatively lower percentage of patients in Greece (13.2%) were offered pharmacotherapy compared with Italy (24.2%) and Netherlands (37.7%).

**Table 7:** **Physician practice patterns by Country**, **CV ASPIRE study**, **Europe**, **2011**

**Characteristic**	**Greece (N=571)**	**Italy (N=443)**	**Netherlands (N=425)**	**Total (N=1439)**
At this visit, was this patient provided with smoking cessation advice? n (%)	499 (87.9%)	323 (73.2%)	303 (71.5%)	1125 (78.5%)
At this visit, was any behavioral support offered for helping quit smoking? n (%)	319 (56.5%)	194 (43.8%)	215 (50.7%)	728 (50.8%)
At this visit, was any pharmacotherapy offered for helping quit smoking? n (%)	74 (13.2%)	107 (24.2%)	160 (37.7%)	341 (23.9%)

## Discussion

To our knowledge, CV-ASPIRE was one of the largest cross-sectional surveys of its kind to explore health risks and prevalence of comorbidities in smokers in the primary care setting and where GPs in Europe were surveyed on issues concerning patient behavior and physician attitudes toward smoking cessation. As expected, we found both patient behavior and physician attitudes toward smoking cessation to vary by country. A possible explanation for these apparent differences in practice patterns could perhaps include differences in the attitude towards the role of GPs in influencing smoking cessation. Differences in the pattern of practices and the negligence of prevention in primary care seem to be the explanation for this finding in some countries [26]. These results are consistent with previous findings which showed varying rates of GP advice on smoking cessation across Europe [18,19,22,23].

A total of 49% of smokers reported at least one COPD related disorder, however only 20% of were reported as diagnosed with COPD. According to the Global Initiative for Chronic Obstructive Lung Disease COPD reference guide, “A diagnosis of COPD should be considered in any individual who has dyspnea, chronic cough or sputum production, and/or a history of exposure to risk factors for the disease, especially cigarette smoking” [27]. This finding suggests that COPD may be under diagnosed in smokers.

In Greece and Italy, the primary reason for consultation with their GP was general medical examination (57%); this was notably higher than in the Netherlands (16%), where the primary reason for consultation was cardiovascular related (32%). One of the factors that may help explain this is that more patients may be visiting their GP in Italy and Greece in order to obtain a repeat prescription. In rural Greece it has been shown that over half of patients visit their GP solely to ask for a prescription, and 70% of prescriptions are renewal of an older one [28].

Interestingly, the perception of GPs and patients differed with regards to the types of smoking advice offered to smokers in the primary care setting. For instance, a higher proportion (73%) of Italian patients reported that their GPs offered smoking advice compared to Italian GPs themselves (48%). The referral for behavioral support and pharmacotherapy was reported in the 3 participant countries consistently lower by patients than by GPs. Perhaps the difference in reporting rates could be explained in part by recall bias where GPs more readily remember the delivery of service rather than their failure to offer the service. They could also be explained through prevarication bias such that GPs could be more inclined to state that they offered the advice to their patients while patients are more inclined to deny that such advice was ever offered to them.

Demographic characteristics were for the most part distributed similarly by country (Table 3).

Various prediction models were employed in this study to estimate the absolute risk of outcomes previously established to be associated with smoking. A hypothetical population of non-smokers, derived directly from the prediction model (by setting smoking status to non-smoking), was applied in these models. This approach allowed us to mimic a counterfactual scenario where an identifiable cohort of smokers could be classed as non-smokers. The main advantage of generating a hypothetical population of non-smokers from a prediction model is that it avoids the need for sampling non-smokers into the study. Moreover, it also provides control of all confounders included as part of the prediction model since the prediction model assumes that only those variables included in the model are relevant for the prediction of events.

A limitation of this approach, however, is that the model may underperform in the real-world setting given that any known confounding factor not included in the prediction model or an unknown confounding factor may influence outcomes. For instance, the SCORE model only considers age, gender, smoking status, systolic blood pressure, and lipid levels to be important in the prediction of death due to cardiovascular disease and ignores potential important covariates such as alcohol use, family history, diet, exercise, and concomitant medications. Moreover, these prediction models do not provide complete information about what might have happened if a long-term smoker was to quit smoking, since the models themselves do not account for past exposure to smoking and its potential effects on future outcome. Another limitation, is that the simulated non-smokers have identical blood pressure and lipid levels as the smokers. It has been found that smokers tend to have lower HDL [3] and higher blood pressure [29] than non-smokers. Since the simulation is not accounting for this, the results may be considered a conservative estimate of the impact on CV risk of smoking.

Despite these limitations, the simulated population of non-smokers provided a convenient population from which to derive the relative risk attributable to smoking for cardiovascular events. The relative risk was reported in this study because it quantifies the risk in a clear and interpretable percentage that can easily be related to the impact of smoking on cardiovascular events.

The prediction models found expectedly that smokers experienced a quantitatively higher level of risk in each of the cardiovascular event categories considered when compared to a hypothetical population of non-smokers sharing the same risk factor profile. The directionality of the association was consistent (i.e., smokers were always found to be at higher risk) regardless of the prediction model. We were able to predict, for instance, through the SCORE model that this study population of smokers have a 100% increased probability of death due to cardiovascular disease in the next 10-years compared to non-smokers. The relative risk derived using the Framingham models for 10-year risk of coronary heart disease and 10-year risk for “hard” coronary heart disease showed an increase in risk of 50% (from 12% to 18%) and 65% (from 8% to 13%), respectively. The Progetto CUORE model found a similar risk increase of 68% (from 6% to 10%) for coronary or cardiovascular events.

The discrepancy in results across the prediction models just described can be explained by differences in the outcome of interest. None of the scores predicted risks are directly comparable. For example, the SCORE model predicts death due to cardiovascular disease whereas the Framingham coronary heart disease score predicts new cardiovascular events, not necessarily resulting in death. Similarly, the Progetto model predicts the risk of a major cardiovascular event, in comparison to the Framingham coronary heart disease model which predicts the risk of coronary heart disease, and one would not expect all patients diagnosed with coronary heart disease to necessarily experience a major event. Another reason why the model predictions may differ is the underlying population used to derive the models: the Framingham score was derived using a population from the United States; the SCORE model a European and the Progetto an Italian population.

The eligibility criteria for this study was restricted to individuals 40 years of age or over, which consequently may limit the generalizability of results to a younger population or those who have smoked for a shorter duration of time. Subjects were also required to have available information on blood pressure and lipids within the past 12 months of the study. Given that subjects with a history of cardiovascular disease are more likely to have available measures on the above laboratory parameters, we expect that this study population would have a higher prevalence of cardiovascular disease than a general population of smokers. Moreover, the generalizability of this study may be biased towards those at higher risk of CVD.

## Conclusions

The findings of this study reinforce the importance of smoking as a significant predictor of long-term cardiovascular events. The consistency in the elevation of relative risk found across prediction models reinforces the need to reduce and eventually eliminate this important source of morbidity and mortality from the general population through effective smoking cessation programs. Future country-specific interventional strategies for smoking cessation should consider targeting GP practices given the GPs’ role in patient care.

It has been previously shown that smoking is the second most important modifiable risk factor associated with myocardial infarction, after LDL: HDL ratio [30], and the excess risk of experiencing a heart attack is halved within a year of quitting in comparison to continuing smokers [31]. Patients attending GP practices diagnosed with hypercholesterolemia are routinely treated for the condition. However, smokers are far less likely to receive smoking cessation treatment when visiting their GP, there may be an unfounded disparity in treatment for smoking compared to other risk factors. According to the United States Department of Health and Human Services “it is difficult to identify any other condition that presents such a mix of lethality, prevalence…and neglect …despite effective and readily available interventions” [32]. Further analyses may show that one of the best gains in health could be obtained by tackling the most important modifiable risk factors, smoking being one of them. The findings in this study may help future strategies for focusing smoking cessation programs in the primary care setting.

## Abbreviations

CI: Confidence interval; COPD: Chronic obstructive pulmonary disease; CRF: Case report form; CV: Cardiovascular; CVD: Cardiovascular disease; FTND: Fagerström Test for Nicotine Dependence; GP: General practitioner; HDL: High-density lipoprotein; LDL: Low-density lipoprotein; SCORE: Systematic Coronary Risk Evaluation; SD: Standard deviation; SBP: Systolic blood pressure.

## Competing interests

The study was funded by Pfizer. PM and FM are employees at Pfizer. CL, HR and RI received research funding from Pfizer. AB and MN have no competing interests.

## Authors’ contributions

PM conceived study, participated in its design, coordination and data interpretation, lead manuscript development. CL, HR and RI participated in coordination and implementation of the study, contributed to drafting the manuscript. AB performed statistical analysis and drafted manuscript. MN participated in its design and coordination, helped to draft the manuscript. FM participated in its design and coordination, helped to draft the manuscript. All authors read and approved the final manuscript.

## Authors’ information

PM: Senior Medical Manager, Primary Care BU Europe, Pfizer, Walton Oaks, Surrey, KT20 7NS, UK, email: Pablo.Mallaina@pfizer.com. CL: Professor of General Practice and Primary Care, Head of Clinic of the Social and Family Medicine, School of Medicine, University of Crete, Greece, email: lionis@galinos.med.uoc.gr. HR: Doctor of Forensic Medicine, Bennebroek Primary Care Center, The Netherlands, email: roverz@hotmail.nl. RI: Medical Practitioner, Primary Care Varese ASL, distretto di Tradate, Italy, email:renzoimp@libero.it. AB: Statistical Scientist, Real-World and Late Phase Research, Quintiles, Reading, UK, email: Andrew.Burgess@Quintiles.com. MN: Director, Late Phase Biostatistics, Real-World and Late Phase Research, Quintiles, Reading, UK, email: Mark.Nixon@Quintiles.com. FM: Senior Medical Manager, Primary Care BU Europe, Pfizer, Primary Care BU Europe, Walton Oaks, Surrey, KT20 7NS, UK, email: Franco.Mondello@pfizer.com.

## Pre-publication history

The pre-publication history for this paper can be accessed here:

http://www.biomedcentral.com/1471-2458/13/362/prepub
